# Unravelling actionable biology using transcriptomic data to integrate mitotic index and Ki-67 in the management of lung neuroendocrine tumors

**DOI:** 10.18632/oncotarget.27874

**Published:** 2021-02-02

**Authors:** Venkata S.K. Manem, Olga Sazonova, Andréanne Gagné, Michèle Orain, Babak Khoshkrood-Mansoori, Nathalie Gaudreault, Yohan Bossé, Philippe Joubert

**Affiliations:** ^1^Quebec Heart and Lung Institute Research Center, Quebec City, QC G1V4G5, Canada; ^2^Department of Molecular Medicine, Laval University, Quebec City, QC G1V4G5, Canada; ^3^Department of Medical Biochemistry, Molecular Biology and Pathology, Laval University, Quebec City, QC G1V4G5, Canada

**Keywords:** mitotic index, Ki-67, transcriptomics, lung neuroendocrine tumors, pathway analysis

## Abstract

Pulmonary neuroendocrine tumors (NETs) are a heterogeneous family of malignancies whose classification relies on morphology and mitotic rate, unlike extrapulmonary neuroendocrine tumors that require both mitotic rate and Ki-67. As mitotic count is proportional to Ki-67, it is crucial to understand if Ki-67 can complement the existing diagnostic guidelines, as well as discover the benefit of these two markers to unravel the biological heterogeneity. In this study, we investigated the association of mitotic rate and Ki-67 at gene- and pathway-level using transcriptomic data in lung NET malignancies. Lung resection tumor specimens obtained from 28 patients diagnosed with NETs were selected. Mitotic rate, Ki-67 and transcriptomic data were obtained for all samples. The concordance between mitotic rate and Ki-67 was evaluated at gene-level and pathway-level using gene expression data. Our analysis revealed a strong association between mitotic rate and Ki-67 across all samples and cell cycle genes were found to be differentially ranked between them. Pathway analysis indicated that a greater number of pathways overlapped between these markers. Analyses based on lung NET subtypes revealed that mitotic rate in carcinoids and Ki-67 in large cell neuroendocrine carcinomas provided comprehensive characterization of pathways among these malignancies. Among the two subtypes, we found distinct leading-edge gene sets that drive the enrichment signal of commonly enriched pathways between mitotic index and Ki-67. Overall, our findings delineated the degree of benefit of the two proliferation markers, and offers new layer to predict the biological behavior and identify high-risk patients using a more comprehensive diagnostic workup.

## INTRODUCTION

Neuroendocrine tumors (NET) are a heterogeneous group of malignancies, which represent around 23% of all lung cancers [1]. Based on the WHO classification, they are classified as typical carcinoids (TC), atypical carcinoids (AC), large cell neuroendocrine carcinoma (LCNEC) and small cell lung carcinoma (SCLC) [2]. Clinically, TC’s are low-grade neoplasms that are usually cured by surgery and have good prognosis [3, 4]. While, AC’s are low to intermediate-grade tumors with a more aggressive behavior and benefit from multimodality treatment regimens [5–7]. On the other hand, LCNEC and SCLC are high-grade tumors with poor prognosis that are commonly treated with chemotherapy and/or radiation therapy as they usually present at advanced stage [8, 9]. Hence, an accurate diagnosis is required for patients presented with pulmonary neuroendocrine tumors as it will result in substantial differences in their management and prognosis [10]. Conventionally, the classification of neuroendocrine tumors is based on histological characteristics along with the evaluation of mitotic count and the presence of necrosis [2]. Low-grade tumors have morphological features of well-differentiated neuroendocrine tumors. Typical carcinoids have less than 2 mitoses per 2 mm^2^ with lack of necrosis, while atypical carcinoids show between 2 and 10 mitoses per 2 mm^2^ and/or necrosis. On the other hand, SCLC and LCNEC neoplasms constitute poorly differentiated tumors showing high grade cytologic features, extensive necrosis and mitotic count higher than 10 mitoses per 2 mm^2^ [11].

In pulmonary NETs, the Ki-67 antigen has been evaluated for its diagnostic, prognostic and grading implications [12–14]. Ki-67 identifies proliferating cells spanning across all cell cycle phases (G1 to M) [15, 16], and the expression of Ki-67 is proportional to the mitotic index. To date, the mitotic count has remained the only proliferation criterion in the classification of lung neuroendocrine neoplasms in opposition to extra-pulmonary neuroendocrine tumors, which also rely on Ki-67 proliferation rate. However, Ki-67 antigen has been demonstrated to be a strong prognostic indicator in lung NETs [17, 18]. Although the grading system in lung NET is done by identification of the histological features and mitotic count, there are a subset of these tumors that are difficult to classify due to a discordance between morphology and mitotic rate [19, 20, 44]. In this regard, a recent WHO classification scheme mentions that Ki-67 antigen might have a role in classifying lung NETs [2]. Along these lines, a study was carried out on a cohort of 400 pulmonary NETs, where the authors proposed a grading system that integrated mitotic count and necrosis in addition to Ki-67 index [14]. By combining various thresholds of these variables, a grading system (G1 to G3) was generated based on the occurrence of at least two of three parameters meeting the required cut-offs. The authors have shown that the performance of the combined set of these three variables outperformed the performance of each variable in predicting the overall survival of patients. Hence, it is important to implement a grading system that can complement the current guidelines and unravel the inherent biological complexity of lung NETs, which could eventually result in better therapeutic options to treat these patients.

Many studies in the literature have shown that the two proliferation markers, mitotic rate and Ki-67 index are strongly correlated [14, 21–23], however, there has been a paucity of studies on the similarities and differences of mitotic index and Ki-67 at the transcriptomic level. Also, in order to move further with the integrated approach as proposed by Rindi et al. [14], or, to design a molecular subtyping scheme based on a robust proliferation indicator, it is important to investigate if the two markers drive similar or different biological pathways. If the two proliferation markers govern the same biological mechanisms, then integrating the Ki-67 index to the grading system would become redundant. However, if both capture different biological processes, then it would be useful to add Ki-67 as well into the integrated diagnostic framework. Furthermore, more data is needed to investigate the magnitude of benefit of these markers within the histological subtypes. Recognizing this void in the literature, the main objectives of this study were to: i) compare mitotic rate and Ki-67 index using transcriptomic data in neuroendocrine samples, and ii) identify the strongest indicator of the biological behavior among the histological subtypes of neuroendocrine tumors, i.e., carcinoids and LCNEC’s.

## RESULTS

### Patient features

The clinical characteristics of patients in the cohort are presented in Table 1. According to the ‘modified WHO classification’ (see Methods section), 8 cases were diagnosed as LCNECs and 20 as carcinoids on pathology evaluation. The spectrum of mitotic count and Ki-67 values for all the samples are presented in Table 2. The mean mitotic rate was 22.25 (1–128) per 2 mm², and the mean Ki-67 index was 30.47% (1–100%) across all the lung NET samples in the cohort. In the group of pulmonary carcinoids, the average value of mitotic rate was 4.08 mitoses per 2 mm² (1–17 mitoses per 2 mm²) and average value of Ki-67 proliferative index was 8.32% (1-24%). Within the LCNEC group of tumors, the average value of mitotic rate was 67.68 mitoses per 2 mm² (range: 25–128 mitoses per 2 mm²) and Ki-67 index was 85.87% (range: 58–100%).

**Table 1:** Patient characteristics based on ‘modified WHO classification’

	All cases	Carcinoids	LCNEC
Number of patients	28	20	8
Average age at diagnosis (years)	57.92	56	61.25
Average tumor size (mm)	29.62	25.42	39.62
Sex:			
Male	14 (50%)	10 (50%)	4 (50%)
Female	14 (50%)	10 (50%)	4 (50%)
Smoking Status:			
Ex-smoker	19 (68%)	15 (75%)	4 (50%)
Non-smoker	4 (14%)	4 (20%)	0 (0%)
Active smoker	5 (18%)	1 (5%)	4 (50%)
Recurrence:			
Yes	9 (32%)	5 (25%)	4 (50%)
No	19 (68%)	15 (75%)	4 (50%)

LCNEC: large cell neuroendocrine carcinoma.

**Table 2:** Proliferation markers of patients stratified based on the WHO classification of neuroendocrine tumors

	*N*	Mitotic Rate (per 2 mm^2^)	Ki-67 Index (%)
Carcinoids	20	4.09 ± 4.81	8.32 ± 6.78
Typical	10	0.96 ± 0.52	4.97 ± 5.13
Atypical	10	7.21 ± 5.19	11.66 ± 6.77
LCNEC	8	67.68 ± 33.77	85.87 ± 13.32

LCNEC: large cell neuroendocrine carcinoma. SD: standard deviation. Values are means ± SD.

### Neuroendocrine samples

**Figure 1:**
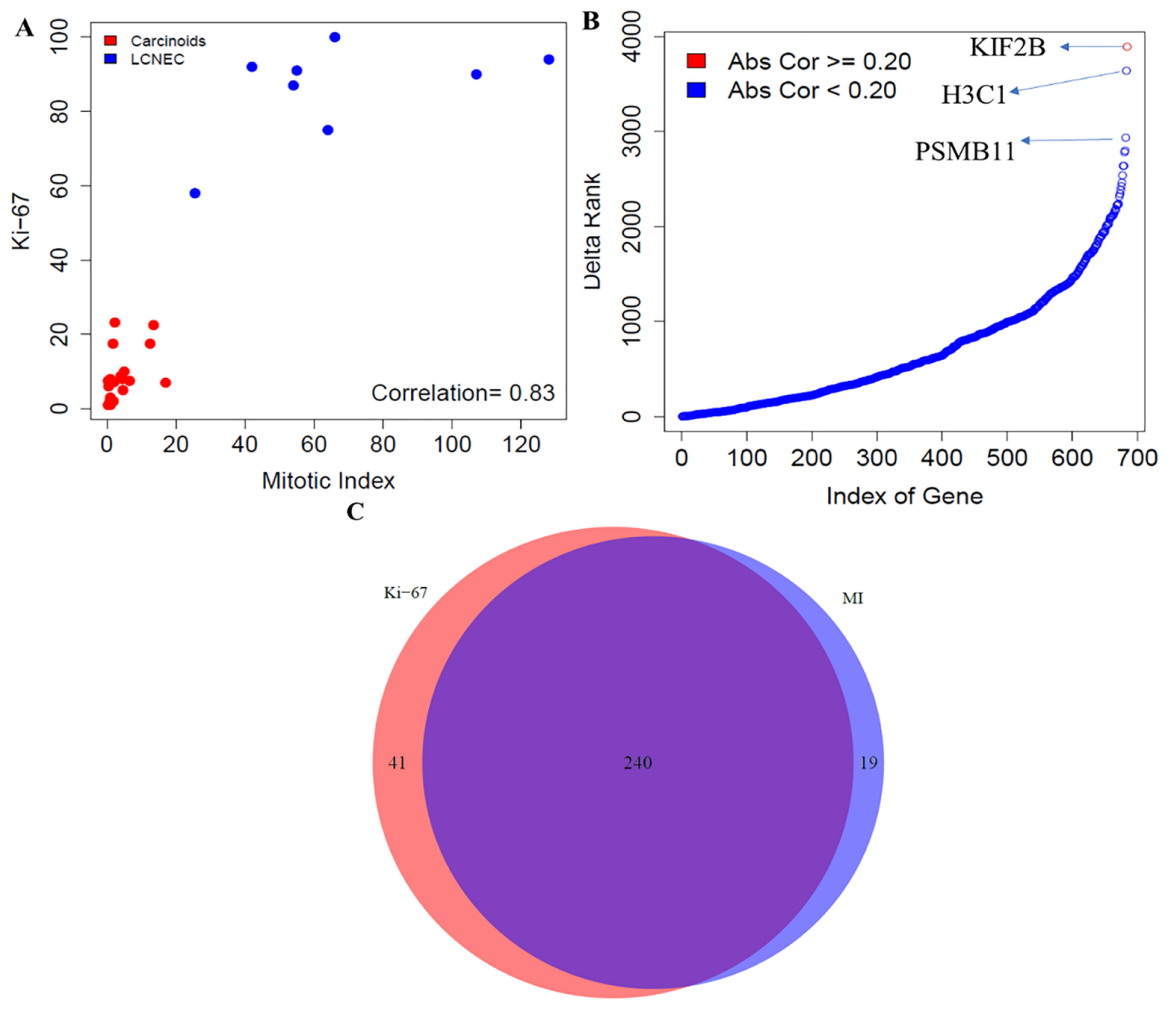
Comparative analysis between mitotic index and Ki-67 in neuroendocrine samples. (**A**) Correlation between mitotic index and Ki-67 using the Spearman correlation. (**B**) Gene level differences - Differences in ranking of genes involved in proliferative pathways; and (**C**) Pathway level differences - Concordance of enrichment of pathways between mitotic index and Ki-67 (FDR < 10%). MI indicates mitotic index.

We performed the comparative analysis between mitotic index and Ki-67 using the gene expression data across all neuroendocrine samples, comprising of both carcinoids and LCNECs. The Spearman correlation coefficient between the two proliferation markers was found to be *r* = 0.83 (Figure 1A). The gene-level differences were then examined by computing the association of genome-wide expression levels with the two proliferation markers (mitotic index and Ki-67) using Spearman correlations. Each of the top 10 positively and negatively correlated genes with mitotic index and Ki-67 were presented in the Supplementary File 1.

In addition, we examined if there were any significant changes in the ranking of strength of correlation of *cell cycle-related genes* with mitotic index and Ki-67. To achieve this, we extracted all the pathways related to cell cycle from the REACTOME database. We searched with the key word, “CELL_CYCLE” and obtained 12 molecular pathways that were associated with cellular proliferation, comprising of 686 unique genes. Large changes in terms of the ranking of strength of correlation of 686 proliferative pathway-related genes with mitotic index and Ki-67 were observed (Figure 1B).

The top three genes that changed the ranking were, KIF2B - a member of the kinesin family that is involved in microtubule activity; H3C1 – a member of the DNA binding protein family that has an important role in gene regulation and cell cycle control; and PSMB11 – a member of proteasome subunit that is presented by major histocompatibility complex I molecules. Overall, our findings revealed that proliferation-related genes were differentially ranked between mitotic index and Ki-67.

Finally, we compared the pathways that correlate with the mitotic index and Ki-67 using all the samples in the cohort. For an FDR < 10%, 259 molecular pathways were enriched using mitotic index, out of which 216 and 43 pathways were positively and negatively correlated with mitotic index, respectively (Figure 1C, Supplementary File 1). For Ki-67, 281 pathways were enriched, out of which 241 and 40 were positively and negatively associated. We found 240 pathways that were common between mitotic index and Ki-67 (Figure 1C, Supplementary File 1). The number of statistically significant molecular pathways that were specific to Ki-67 index and mitotic rate were 41 and 19 respectively (Figure 1C). Quantitatively, we found slightly greater number of pathways that were enriched by Ki-67 (281 pathways) compared to mitotic index (259 pathways). However, we found that a majority of these pathways (241 of them) overlapped between the two proliferation markers, mitotic index and Ki-67. To summarize, our findings suggest that mitotic index and Ki-67 were strongly correlated, and both these markers also drive a similar set of transcriptional pathways underpinning the cell cycle kinetics in neuroendocrine malignancies.

### Subtype-specificity

Herein, we investigated which of these proliferation markers is more reliable and strongest indicators of the biological behavior among the histological subtypes of neuroendocrine tumors. To achieve this, we characterized the biological properties by comparing mitotic index and Ki-67 that favor the two neuroendocrine subtypes – carcinoids and LCNEC, using gene expression data.

#### Carcinoids

**Figure 2:**
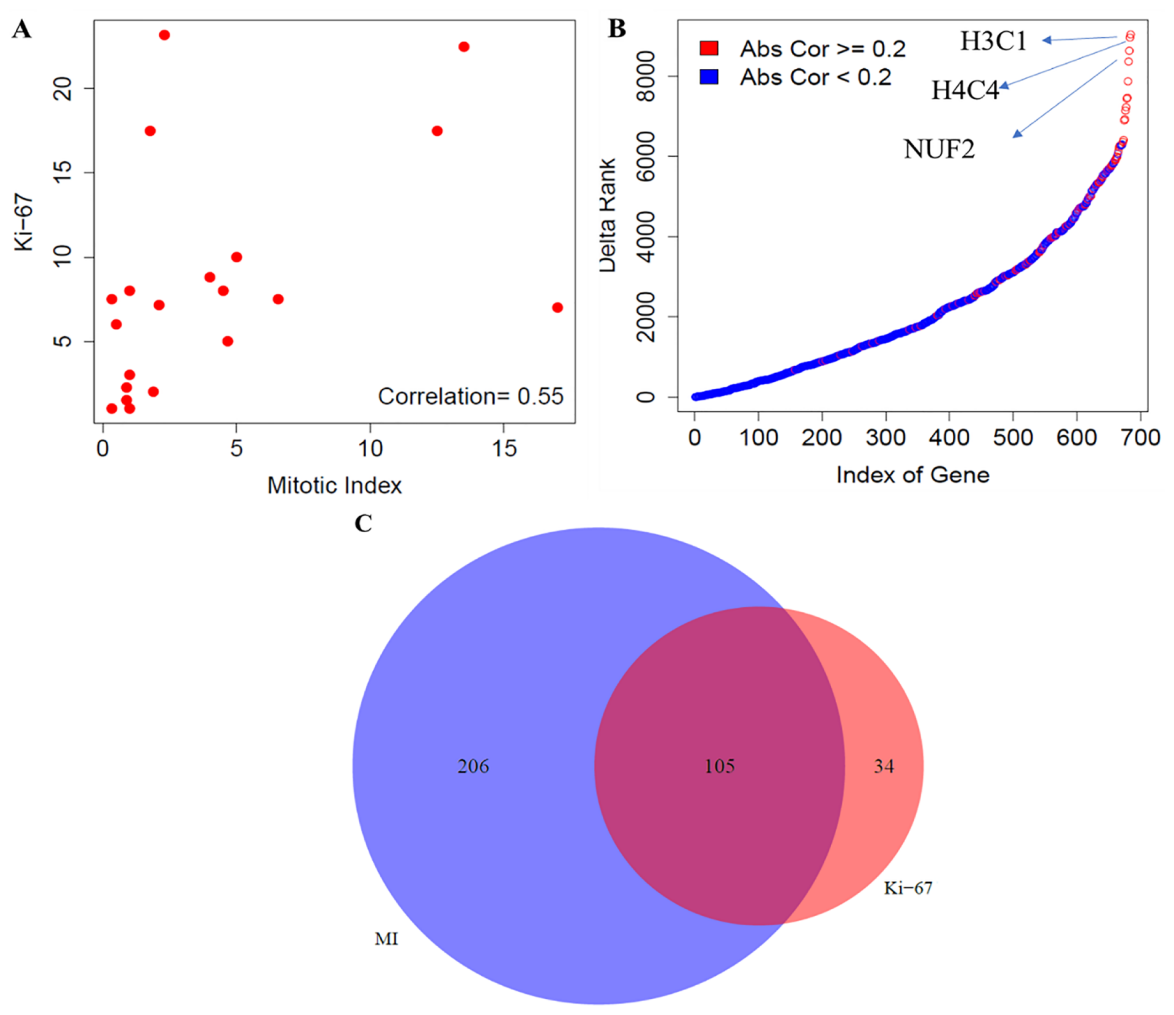
Comparative analysis between mitotic index and Ki-67 in carcinoids. (**A**) Correlation between mitotic index and Ki-67 using the Spearman correlation. (**B**) Gene level differences- Ranking differences in genes related to the proliferative pathways; and (**C**) Pathway level differences- Concordance of enrichment of pathways between mitotic index and Ki-67 (FDR < 10%). MI indicates mitotic index.

The comparative analyses of mitotic index and Ki-67 in this section was carried out using the gene expression data for the low-proliferative group of tumors (*n* = 20). We first examined the association between the two proliferation markers, using the Spearman correlation coefficient, and found a moderate correlation between them (*r* = 0.55) (Figure 2A). Next, we investigated the gene-level differences using the transcriptomic data. To do this, we evaluated the association of genome-wide expression levels measured in carcinoids with the two proliferation markers (mitotic index and Ki-67) using the Spearman correlation. Each of the top 10 positively and negatively correlated genes with the two proliferation markers, mitotic index and Ki-67 were presented in the Supplementary File 1. In addition, we also found large changes in terms of the ranking of strength of correlation with their absolute difference of 686 proliferative pathway-related genes with mitotic index and Ki-67 (Figure 2B). The maximum and minimum difference in correlation was found to be 0.44 and 0.07, respectively. The top three genes that changed the ranking drastically (H3C1, H4C4) belong to the histone family, which play a crucial role in transcription regulation, DNA repair, DNA replication and chromosomal stability; and NUF2 – modulates the cellular proliferation through the control of cell cycle.

Finally, we compared the biological pathways that were associated with the mitotic index and Ki-67. For an FDR < 10%, 311 molecular pathways were enriched using mitotic index, out of which 97 and 214 pathways were positively and negatively correlated with mitotic index, respectively (Figure 2C). Similarly, using Ki-67 as the proliferation indicator, 139 pathways were enriched, out of which 89 and 50 were positively and negatively associated with the Ki-67 index. We found 105, 34 and 206 molecular pathways that were common, specific to Ki-67 index and specific to mitotic rate, respectively (Figure 2C, Supplementary File 1). To summarize our findings, we found a moderate correlation between mitotic index and Ki-67 and observed large changes in the ranking of proliferation-related genes. Furthermore, our analysis demonstrated that mitotic index provides a more comprehensive portrait of the biological pathways compared to Ki-67 among the low to intermediate proliferative carcinoid tumors.

#### Large cell neuroendocrine carcinomas

**Figure 3:**
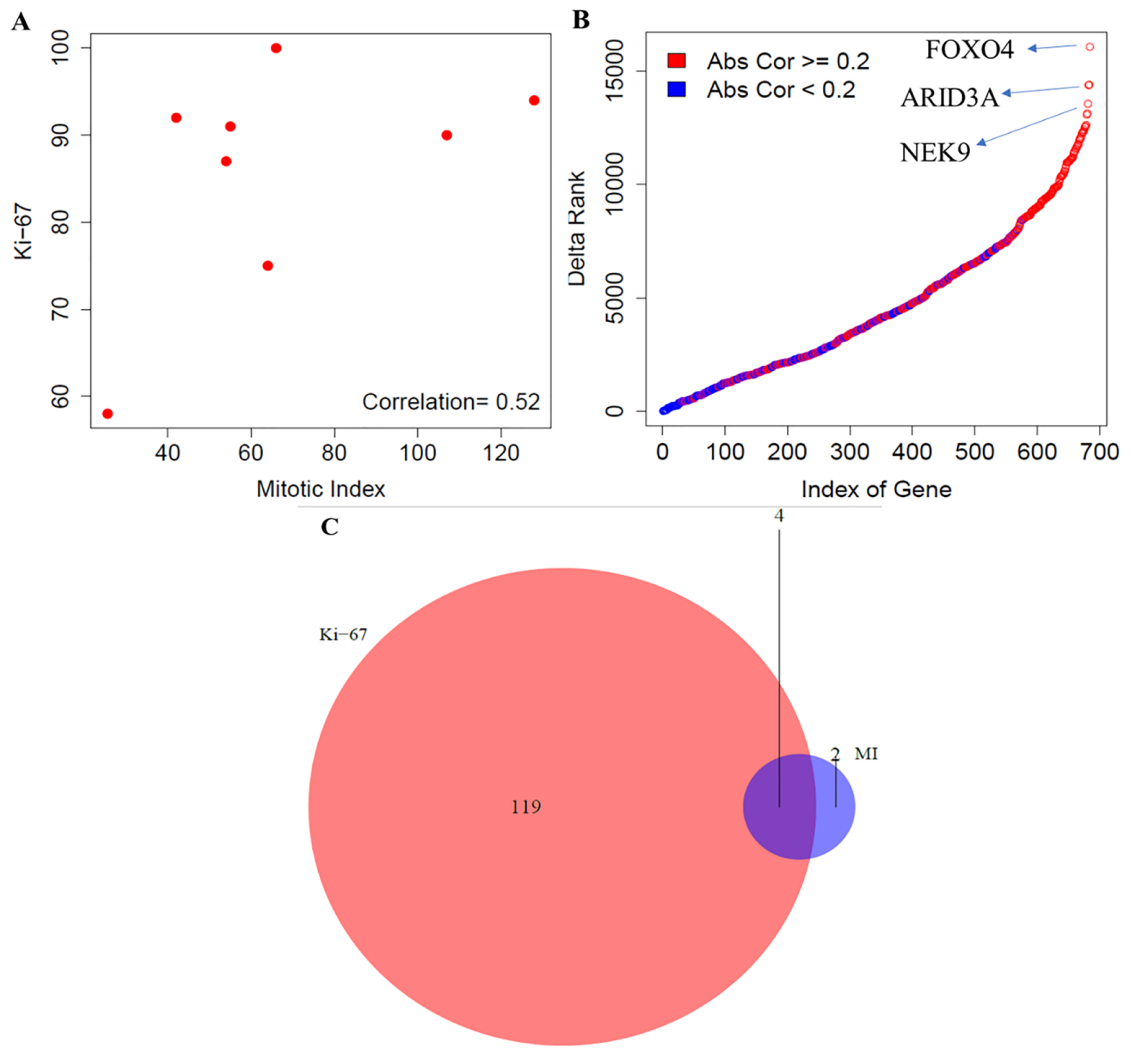
Comparative analysis between mitotic index and Ki-67 in LCNEC. (**A**) Correlation between mitotic index and Ki-67 using the Spearman correlation. (**B**) Gene level differences - Ranking differences in genes involved in proliferative pathways; and (**C**) Pathway level differences - Concordance of enrichment of pathways between mitotic index and Ki-67 (FDR < 10%). MI indicates mitotic index.

In this section, we performed the comparative analysis between mitotic index and Ki-67 using the gene expression data for the high-proliferative group of tumors. We used the Spearman correlation to compute the association between the two proliferation markers and found a moderate correlation, *r* = 0.52, between them (Figure 3A). Gene-level differences were investigated by computing the association of genome-wide expression levels measured in LCNEC with the two proliferation markers (mitotic index and Ki-67) using the Spearman correlation. The top 100 genes correlated with mitotic index and Ki-67 were presented in the Supplementary File 1. We also found large changes in terms of the ranking of correlation strength along with their absolute difference of the 686 proliferative pathway-related genes with mitotic index and Ki-67 (Figure 3B). The top three genes that changed the ranking drastically were FOX04, ARID3A – belongs to the family of DNA binding proteins, which have an important role in cell lineage gene regulation and cell cycle control; and NEK9 – belongs to the family of serine, or threonine protein kinases, which is activated in mitosis and mediates cellular processes that are essential for interphase progression. The maximum absolute difference in the correlation strength was found to be 0.85.

Next, we compared the biological pathways that correlate with the mitotic index and Ki-67 among the high-grade carcinoma group of tumors. For an FDR < 10%, 6 molecular pathways were enriched using mitotic index, out of which 6 and 0 pathways were positively and negatively associated with mitotic index, respectively (Figure 3C, Supplementary File 1). Similarly, using Ki-67 as the proliferation indicator, 123 pathways were enriched, out of which 119 and 4 were positively and negatively associated with the Ki-67 value. We found 4 pathways that were common between mitotic index and Ki-67. The number of molecular pathways that were specific to Ki-67 index and mitotic rate were 119 and 2 respectively (Figure 3C). To summarize our analyses, we found a moderate correlation between mitotic index and Ki-67 and observed larger variations in terms of the ranking of proliferation genes, compared to carcinoid group. Additionally, our findings also indicate that Ki-67 when compared with mitotic index was able to capture more transcriptional pathways among the LCNEC’s.

### Analysis of leading-edge genes

**Figure 4:**
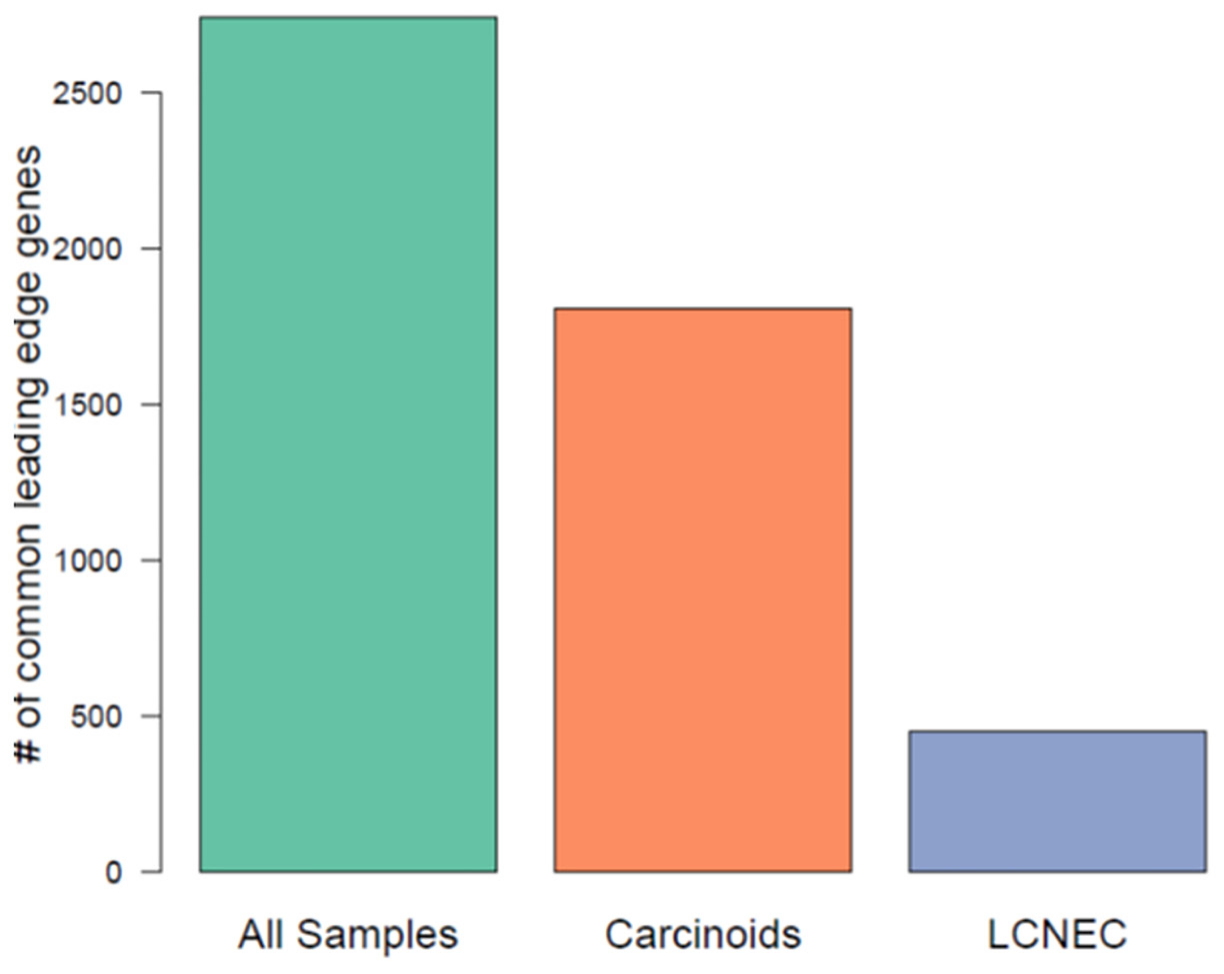
Comparison of leading-edge gene sets among commonly enriched pathways using mitotic index and Ki-67, across all neuroendocrine samples, carcinoids, LCNEC.

To identify which genes in the enriched pathways were driving their enrichment, we performed the leading-edge gene analysis. To achieve this, we focused on pathways that were commonly enriched between mitotic index and Ki-67 within each histological group, and in all neuroendocrine samples separately. Leading-edge genes were extracted from 105, 4 and 240 common pathways that were enriched between mitotic index and Ki-67 in carcinoids, LCNEC and in all neuroendocrine samples, respectively. The intersection of the unique leading-edge gene sets between mitotic index and Ki-67 for each histological group and for all samples is presented in Figure 4. We found a greater number of leading-edge genes that were overlapping in all samples (that has both low- and high- proliferative tumors), suggesting that the enrichment signal is driven by similar set of genes with mitotic index and Ki-67. Within the histological groups, we found a minimal overlap in the leading-edge genes, indicating that different sets of genes drive the enrichment signal with the two phenotypes of interest (mitotic rate and Ki-67).

## DISCUSSION

Lung neuroendocrine tumors represent a heterogeneous group of malignancies with distinct morphological patterns. One of the most important characteristics of cancer cells is their ability to proliferate in an abnormal manner without any external stimuli [36]. The proliferation capacity of cancer cells is traditionally assessed by either counting the mitoses per mm^2^ or through the immunohistochemical analysis of Ki-67. Both mitotic index and Ki-67 have been used to analyze their diagnostic and prognostic value in several tumor types, such as breast, melanomas and non-small cell lung cancers [37, 38, 41, 42, 43]. Recent studies have demonstrated the prognostic role of Ki-67 index in lung neuroendocrine tumors with regards to their overall survival [46–48]. To date, no single variable is sufficient to predict the clinical behavior of some tumors [39, 40], and mitotic count remained the only proliferation marker in classification of lung NETs. Furthermore, predicting biologically distinct NET subtypes along with their clinical outcome remains uncertain, due to the sub-optimality of the mitotic index [44]. Based on this, it is crucial to examine the association of mitotic index and Ki-67 using gene expression data, which can aid in developing an integrated diagnostic framework as well as aid in predicting the biological behavior of these malignancies. In this study, we employed a *de-novo* discovery approach to rigorously investigate the association of mitotic index and Ki-67 at gene- and pathway-level using transcriptomic data in lung NET malignancies.

For this study, we selected 28 lung NET patients consisting of carcinoids and large cell carcinomas. The proliferation capacity of cancer cells was assessed through two markers, namely, mitotic index and Ki-67 antigen for all the samples. Firstly, we found a strong Spearman correlation between the two proliferation markers (*r* = 0.88). Secondly, we found gene-level differences between both the markers, and specifically, the cell-cycle related genes tended to exhibit large variations in the ranking of their correlation strength.

In addition, we hypothesize that the cell cycle related genes could potentially be used to characterize the differences between the two proliferation markers using transcriptomic data, however, these findings warrant further investigation. Thirdly, pathway analysis revealed that both mitotic rate and Ki-67 elicits similar set of pathway responses underpinning the cell cycle kinetics of neuroendocrine malignancies. Finally, we found a large overlap in the leading-edge gene sets among the common pathways that were enriched between mitotic index and Ki-67. While our study uncovered the differences and similarities between mitotic index and Ki-67, these findings primarily exemplify the calculable role of these two markers at the transcriptomic level within the lung NET patients. The outcomes of our study suggest that integrating mitotic index and Ki-67 markers into the diagnostic framework could potentially be redundant, since both these markers govern similar set of biological mechanisms. However, this will require further validation involving larger cohorts to determine their independent and additive value to achieve maximal diagnostic results, which can help in stratifying lung NET subtypes more effectively, like other cancer types [14, 45].

Given the disease heterogeneity of lung NET malignancies, it may be difficult to delineate the true extent of benefit of mitotic index and Ki-67 within the histological groups. To investigate this, we examined the association of mitotic index and Ki-67 with the expression profiles across two distinct lung NET subtypes – carcinoids (*n* = 20) and LCNEC (*n* = 8). We found moderate correlation (*r* = 0.55, *r* = 0.52) between mitotic index and Ki-67 in carcinoids and in LCNEC, respectively. We observed larger variations in terms of the ranking of cell cycle-related genes in both the subtypes, while the variations were found be larger in the LCNEC group. We hypothesize that these set of proliferation-related genes could potentially be used to characterize the differences or similarities between the two proliferation markers using gene expression data. Using pathway analysis, we were able to show that there is considerable variation in the molecular pathways that were associated with mitotic rate and Ki-67 index across carcinoids and LCNEC. While mitotic index provided a more comprehensive characterization of molecular pathways compared to Ki-67 in carcinoids, Ki-67 elicited a broad characterization of biological pathways compared to mitotic index in the LCNEC group. This reflects that specific pathway properties can explain the inherent biological complexity of these subtypes. Furthermore, to better understand which genes tend to be drive the commonly enriched pathways between mitotic index and Ki-67, we capitalized on the leading-edge genes of a pathway. We found distinct sets of leading-edge genes that drive the biological pathways underpinning the cell cycle kinetics defined by mitotic index and Ki-67 in both the subtypes. Altogether these observations suggest that depending on the histological subtype, either mitotic index or Ki-67 can be used to discover the inherent biological complexity and diversity of these malignancies.

Despite the relative merits of mitotic index and Ki-67, and the degree of correlation between these two biomarkers in different cancer types, less attention has been paid to integrate these two biomarkers in lung NETs. To summarize our findings, we found that mitotic index and Ki-67 markers govern similar set of biological mechanisms (at the pathway-level). Therefore, we hypothesize that integrating both these proliferation indices into the classification framework could potentially be redundant in stratifying lung NET subtypes more effectively. In order to translate these findings into a clinical setting, we will require further validation involving larger cohorts to determine their additive or independent value in a classification setting, which is the focus of future investigations. With regards to the histology-based findings, we hypothesize that either mitotic index and Ki-67 could potentially offer a novel characterization of the biological behavior and identify patients who will benefit from a multi-modal therapeutic regimen. The rational integration of these mutually inclusive proliferation variables within the lung NET subtypes could strengthen the current practices to improve clinical decision making for biologically diverse malignancies. Although our findings are of significant interest from a biological point of view, there were some limitations, which include, *i)* Sample size: due to small sample size of the LCNEC group, the gene-level and pathway-level associations with mitotic rate and Ki-67 index is less robust; *ii)* Inter-observer variability: there is a considerable interobserver variation in the histopathological characterization of lung NET’s, i.e., in samples with low to medium values of mitotic index [12]. The current study forms a basis for further validation on larger datasets that have transcriptomic data, mitotic index, and Ki-67, which will be the focus of our future investigations.

## MATERIALS AND METHODS

### Study design

The study was performed on the cohort from a single pulmonary pathology reference center with the approval of the institutional ethics committee (#21045). Pulmonary resected cases of lung neuroendocrine tumors diagnosed between 2000 and 2016 were reviewed. These samples were part of the Quebec Respiratory Health Network Tissue Bank (https://rsr-qc.ca/biobanque/) at the Institut universitaire de cardiologie et de pneumologie de Québec (IUCPQ). The cohort included a total of 28 lung neuroendocrine cases. Among these patients, 10 samples were typical carcinoids, 7 samples were atypical carcinoids and 11 cases were LCNEC based on the 2015 WHO classification. The availability of frozen tissue along with the quality and the amount of extracted RNA were assessed. For carcinoids, we selected the cases such that the spectrum of their mitotic index, i.e., of 0–10 mitoses per 2 mm² is covered. All the original H&E slides were reviewed by two thoracic pathologists to confirm the diagnostic, including assessment of the mitotic count and the Ki-67 index. We used the WHO guidelines to classify the tumors, except for cases with 10–20 mitoses per 2 mm^2^, as we and other studies have shown that they usually or may exhibit carcinoid-like morphology [28–31, 44]. Throughout the manuscript, this will be termed as the ‘modified WHO classification’. Along these lines, we carried out the re-evaluation of these samples, and showed that a subset of LCNEC tumors displayed features consistent with carcinoid samples using the histological features, IHC profiles and transcriptomic data. Among the 11 LCNEC samples in our cohort, three samples that had the mitotic rate between 10–20 mitoses per mm² were reclassified as atypical carcinoids. To summarize our cohort, based on the ‘modified WHO classification’, the number of patients presented with carcinoids and LCNEC were 20 and 8, respectively.

### Histopathological, mitotic rate and Ki-67 evaluation

The histological analysis and the mitotic rate evaluation of tumors were carried out based on the 2015 WHO criteria [11]. Histological features such as the distinct morphological patterns, extent of necrosis and cytological nuclear features were recorded. The mitotic counts were assessed on H&E slide and expressed as the number of mitoses per 2 mm² in the most mitotically active areas and tumors with less than 10 mitoses per 2 mm² were classified as carcinoids. In addition, the proliferation index was assessed using Ki-67 immunohistochemistry (clone MIB1 from Dako, Dako-Agilent Technologies). One tissue block for Ki-67 immunohistochemistry was randomly chosen for each case as compared to multiple blocks used for mitotic counts on H&E stained slides. Ki-67 calculation was done manually by counting the percentage of cells with positive nuclear labelling. Briefly, 500 tumor cells were evaluated in the hot-spot region. The highest Ki-67 value was considered for the hot-spot, while the average value of Ki-67 was calculated as the mean across the slides.

### RNA extraction and library preparation

Tumor tissues were collected at time of the surgery, with an ischemic time of less than 30 minutes. Using RNeasy Plus Universal Mini kit, the RNA was extracted from 30 mg of frozen lung. The concentration and purity of RNA were verified by UV 260/280 nm ratio, and the quality of RNA was checked using a TapeStation 2200. In order to prepare RNA sequencing libraries, the Illumina TruSeq stranded Total RNA library prep kit with Ribo-Zero Gold was used. Briefly, 10 μg of the total RNA was used. The RNA was fragmented, which was then used as a template for cDNA synthesis. This cDNA was then converted into double stranded DNA, which was then end-repaired to incorporate the index adaptor for multiplexing. Following the purification, 15 cycles amplification was performed using a polymerase unable to incorporate dUTP, and hence, the second strand was quenched during amplification. The quality of final amplified libraries was then examined with a DNA screen tape D1000 on a TapeStation 2200. Subsequently, the RNA-seq libraries were sequenced on an Illumina HiSeq 2500 V3 system for paired-end 100 bp sequencing [44]. More details about the library preparation can be found in our earlier study [44].

### Processing of RNA-seq and data analysis

We performed the processing of the RNA-seq data using the Kallisto pipeline with the default analysis settings and parameters [24, 44]. The gene expression was computed from RNA-seq data to quantify expression using a pseudo-alignment method. Ensembl GRCh38 reference transcriptome was used for the workflow, and gene transcripts mapped data were normalized to TPM (Transcript Per Million). The quantification was carried out on the full transcriptome and through summing the transcript-level TPM values, we calculated the gene-level TPM-values. The expression values (of 19095 protein coding genes) were calculated using the log2(TPM+1) that was used for all the downstream analyses in this study.

### Association of gene expression with mitotic rate and Ki-67

We performed a univariate association between gene expression levels and the two proliferation markers (mitotic index or Ki-67) using Spearman correlation. This analysis was done to compare and highlight significant genes that are positively and negatively associated with proliferation markers in carcinoids, LCNEC and in all the samples. This analysis would enable us to identify top genes that changed their ranking in terms of the correlation strength with the two proliferation markers.

### Gene set enrichment analysis

The pathway enrichment analysis on the gene expression data was carried out using the gene set enrichment analysis (GSEA) methodology [25] with transcriptional pathways defined by the REACTOME dataset (version 7) that is a part of the Canonical Pathways sub-collection (C2) from the molecular signatures database (MSigDB), consisting of 1499 distinct molecular pathways. Genes were ranked based on their coefficient of correlation between the gene expression and the proliferation marker scores (mitotic rate or Ki-67 index). GSEA was then used to compute the enrichment score for each pathway, and the statistical significance was calculated using a permutation test (10000 permutations) as implemented in the piano *R* package [26]. Nominal *p*-values obtained for each molecular pathway were corrected for multiple testing using the false discovery approach (FDR) [27], with p.adjust function in the base *R* package.

### Leading-edge gene analysis

To analyze if the same set of genes were driving a pathway enriched by both mitotic index and Ki-67, we performed the leading-edge gene analysis. GSEA method returns a subset of genes, termed as the leading-edge genes, which drives the enrichment statistic in the pathway analysis. The leading-edge genes were obtained from the enrichment score that is defined by the maximum deviation from zero. This set of leading-edge genes are considered to be of high biological interest due to appearing at higher frequencies among the pathway subsets [35], which can also be used to build gene signatures [32, 33] or subtyping classifiers [34]. We extracted the leading-edge genes from the pathways that were commonly enriched between the two phenotypes of interest, mitotic index, and Ki-67 within the carcinoids, LCNECs and in all neuroendocrine samples. Within each group, the extracted leading-edge gene sets were compared using the Venn diagram, an R package.

## SUPPLEMENTARY MATERIALS





## Abbreviations

NET: Neuroendocrine tumors
LCNEC: Large cell neuroendocrine carcinoma
GSEA: Gene set enrichment analysis
