# Addendum: Unravelling actionable biology using transcriptomic data to integrate mitotic index and Ki-67 in the management of lung neuroendocrine tumors

**DOI:** 10.18632/oncotarget.28321

**Published:** 2022-12-06

**Authors:** Venkata S.K. Manem, Olga Sazonova, Andréanne Gagné, Michèle Orain, Babak Khoshkrood-Mansoori, Nathalie Gaudreault, Yohan Bossé, Philippe Joubert

**Affiliations:** ^1^Quebec Heart and Lung Institute Research Center, Quebec City, QC G1V4G5, Canada; ^2^Department of Molecular Medicine, Laval University, Quebec City, QC G1V4G5, Canada; ^3^Department of Medical Biochemistry, Molecular Biology and Pathology, Laval University, Quebec City, QC G1V4G5, Canada

**This article has an addendum:** All human tissues were sourced from the IUCPQ Biobank, which includes only patients that have signed a consent form for research.

Original article: Oncotarget. 2021; 12:209–220. 209-220. https://doi.org/10.18632/oncotarget.27874

