# Supplementary material for: Unravelling actionable biology using transcriptomic data to integrate mitotic index and Ki-67 in the management of lung neuroendocrine tumors
Source: Oncotarget. 2021 Feb 2;12(3):209–20. doi: 10.18632/oncotarget.27874 (PMC7869577; doi:10.18632/oncotarget.27874)
Supplement: Supplementary file 1 [file oncotarget-12-209-s001.pdf]

Unravelling actionable biology using transcriptomic data to integrate mitotic index and Ki-67 in the management of lung neuroendocrine tumors

SUPPLEMENTARY MATERIALS

Supplementary File 1: Gene differences and pathway differences among neuroendocrine samples, carcinoids and LCNEC. See Supplementary File 1
